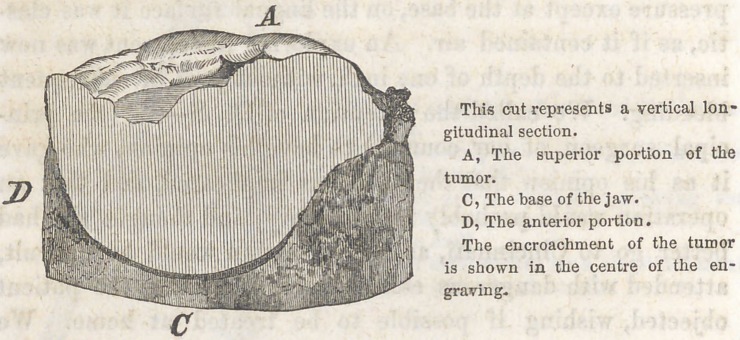# Miscellaneous

**Published:** 1858-09

**Authors:** 


### Miscellaneous.

### A CASE OF FRACTURE OF THE LOWER JAW, AT ITS NECK.

BY M. O. HEYDOCK, M. D., CHICAGO, ILL.

I was called upon, the evening of the 21st of May, to see Mrs. B. F. H., who had, I was told, just been thrown from her carriage, and severely injured. Upon my arrival I found that a fracture of the lower jaw constituted the only injury of a serious nature: the treatment of which is the subject of this paper.

The fracture was evidently at the base of the left condyle, crepitus was very marked and distinct, and deformity arose from a swaying of the jaw to the left, owing, as I suppose, to the contraction of the pterygoid muscle. The fracture was easily reduced, the ordinary roller bandage applied, and cold water dressings ordered for the night. Upon calling the next morning, I found, that soon after falling asleep, displacement had taken place, and it was in the same condition as at first.

I now applied a pasteboard mould and the roller as securely as possible. The next morning I found that this had served the purpose during the day, yet during the sleep, displacement had again occurred, as in the previous night.

I now, with the approval of Dr. Freen, who had seen the case with me, had a spring made, partially encircling the neck, having a pad at each extremity, making pressure upon the left ramus, and over the right articulation, hoping to counteract and overcome the action of the pterygoid muscle.

This acted indifferently well, and was thrown aside. I now used one after another, starch, binder's board, and straps, and during the ensuing ten days, almost every bandage I could find, approved by authors, or suggested by friends. But each and every one failed me, in my effort to retain the jaw in its place, during the night, when voluntary control was lost in slumber. Two weeks had elapsed, displacement had occurred each and every night, crepitus was still as marked as ever, and pain, on motion as great.

The prospect was anything but encouraging, a false joint seemed not only a possibility, but a probability, unless by some continuance, immobility could soon be obtained. I had exhausted my inventive recourses in external appliances, and now made a careful examination of the teeth, to see if from them I could not obtain some hint, which would assist me to accomplish the object I had in view. I observed that the upper central incisors were widely separated, and it occurred to me that advantage might in some way be taken of this peculiarity. A few hours elaborated the idea which had presented itself, and which was, with the modification to be hereafter mentioned, successfully carried out and executed.

One idea suggested was, that a mould of the teeth of the lower jaw should be taken, running as far back as the molars, and from this a gold cap should be made, which should snugly fit, and be securely and firmly attached to the teeth by clasps or otherwise. To this cap, at the point of separation between the central incisors spoken of above, a fragment of gold was to be attached, which would, when the jaws were closed, pass up between these incisors, somewhat in the manner of a wedge. If I am understood, it will be seen that if the cap retains its place, I have by the wedge-like process, overcome the tendency te lateral displacement.

I now called upon Dr. Allport, a Dentist of our city, stated my project to him. He suggested that the pressure of the wedge, even though slight, and though not exerted during the day, while the jaw was under the control of the patient, might give rise to some soreness and irritation, if continued as long as the nature of the case demanded. He proposed to overcome this objection, by fitting a cap for the upper jaw, similar to that of the lower, into which the wedge should be inserted, thus distributing the pressure. The two caps were then to be secured together by a couple of slender bars, crossing from one cap to the other.

Impressions were now taken in wax, and the caps made after the same manner as the plates made by Dentists for artificial teeth. Upon placing the caps in position, it was found as we anticipated, that the gold, operating as a foreign body, would not admit of a closure of the jaws. To obviate this, the opposing and infringing surfaces were cut away, exposing the crowns of all the teeth; thus permitting a close approximation of the jaws, and at the same time furnishing an outlet for the secretions which would naturally accumulate within the caps, and prove a source of annoyance and irritation.

The relation one cap would bear to the other was ascertained, by placing them in position and directing the patient to bite into a mass of softened wax, and while the caps were embedded in the wax, they were removed and secured in this relation, by bars crossing in the neighborhood of the canine teeth of each side.

Dr. Allport now suggested that a thin layer of gutta percha should be placed inside the caps, which might, by its presence as a lining, prove less irritating than the plates alone, while at the same time it would more perfectly adapt itself to the inequalities of the surfaces, than the metal. This, after being softened in hot water, was placed within the caps, and then placed "*in situ*," while it was soft and pliable, and as hot as could well be borne. The patient was then directed to shut the jaw naturally, and it was then firmly pressed home and immovably fixed.

I applied the roller bandage at night for a week or so, to guard against any possibility of displacement from fright or other cause, though there seemed to be little occasion for it, for voluntary motion even was lost to the patient. The natural projection of the upper teeth over the lower, combined with the slight separation of the jaws, caused by the caps, gave ample opportunity for the ingress of soups and slops, by which the patient was nourished and supported until the removal of the caps.

During the first two weeks, considerable pain was experienced in the neighborhood of the fracture, and at the expiration of that time, the provisional callus was perceptible through the tissues. Four weeks having elapsed from the day in which the caps were applied, and six from the date of the fracture, they were removed, and union found to be complete.

For the first few days the articulating surfaces of the teeth did not readily coalesce, but at the end of a fortnight, the recovery was perfect and satisfactory in every particular.

There was, upon removing the caps, some soreness of the mucous membrane, but this rapidly subsided, under the use of an astringent gargle, combined with chlorate of potash.

As a general rule, fractures of the jaw are easily treated, the roller, pasteboard and starch proving sufficient. But in this case they were of no service in restraining the lateral motion, and until this experience, I confess, I was not aware how difficult a thing it was to control that movement. I certainly gave them a fair trial, for Furgusson tells us in his work on surgery, that he "does not take particular pains about the bandages, after the fifteenth day," while in this case two weeks elapsed without any perceptible change for the better; nor was there any promise that there would be, if the same course was continued for a longer period.

In closing, I would say that it is not of course to be expected that in all fractures of this nature, the incisors will be found conveniently separated, and admitting of an application of just such a contrivance as this. But this report will have accomplished its errand, if from it any one shall have obtained a hint, assisting him to control the lateral movementof the jaw. A thing which like many things else untried, seems to the uninitiated simplicity itself, while in fact it gives rise to an anxiety and "vexation of spirits" not dreampt of in their philosophy.

A A represents the two caps.

C C the cross connecting them.

B the wedge passing between the incisors and hidden within the cap.

—*Chicago Medical Journal.*

### REMOVING SUCTION PLATES.

BY MERCHANT KELLY.

After getting the "bite," I remove the wax from a *very* small hole, afterwards soldered, near the center of the chamber of a suction plate, thus admitting air, and causing the plate to be easily displaced, without the usual danger of bending the wax, or changing the impression of the "bite," &c. On page 350, of Volume XI, of Dental Register, J. D. Winter wishes to know if pulverized plaster models would make a good substitute for sand. It appears to be too compact, and not porous enough. Casting made in sand, made adhesive by mixing with it pulverized plaster models, are very smooth, and the sand tough and porous.

---

### A SOCKET HANDLE.

Below we give a cut of a socket handle used by, and brought to the notice of the profession, by Dr. I. Forbes, of St. Louis. The principle is an excellent one, and should have been employed long ago.

It consists of a screw socket, fixed in a suitable handle; into this socket is screwed that portion of the instrument that grasps the point or cutting portion of the instrument. This has two jaws, vice-like in appearance, with a longitudinal groove in each, so that when the jaws are closed, a square hole is formed, for the reception of the small instruments. The jaws are closed firmly together, or upon any thing placed between them, by being screwed into the socket, it being somewhat beveled, so that as it passes in, it is pressed firmly together.

Dr. Forbes uses many different forms of instruments—cutting instruments in particular. He values very highly, cutting instruments of a gouge form, for opening up cavities, and indeed for forming them.

These socket handles are not made by any dental instrument maker, that we are aware of. They should be made, and for sale by our dental depots. The cut represents all parts of the instrument.

A is the handle, which contains the socket. B is the portion that screws into the socket, and grasps the small instrument. C is the small instrument used with it.

Toland will furnish them to the profession. T.

---

### MERRY'S DRILL STOCK.

A is the stationary handle of the instrument.

B is the rotary handle, that by which the drill is turned.

C is the spiral spring connecting the handle with the socket.

D is the drill.

E is the drill socket with its attachment to the permanent handle.

This drill stock will be better understood from our engraving, than it can be from any written or verbal description. The operator has as perfect control of the instrument, as he has of the common straight rotary drill. There is no complex machinery by which the drill is worked. It is turned by the fingers. That portion of the instrument which holds the drill being connected with the handle by a spiral spring, the drill can be made to work in any direction, by simply bending the spring. The drills are inserted into a common "taper socket." But the engraving is the best description we can give.

### EXCISION OF A PART OF THE INFERIOR MAXILLARY BONE.

BY S. F. COMPTON, HILLSBORO', OHIO.

In Nov., 1857, Mrs. G—— of this place, aged 40 years, of spare habit and good health, called my attention to an enlargement of the right side of the inferior maxillary. The tumor was at that time about the size of an almond, and located about half way between the symphisis and angle of the jaw. At this point the teeth had been removed years previous, and no symptoms of disease had ever been manifest until a few months previous to the call; uneasiness and pain was felt some time previous to the appearance of any enlargement; after the tumor began to grow rapidly, no pain was experienced except occasional lancing pains, which was not severe and only continued a moment. The tumor was quite hard, unyielding to pressure except at the base, on the lingual surface it was elastic, as if it contained air. An exploring instrument was now inserted to the depth of one inch, without pain or subsequent bleeding. We called the attention of Dr. S—— (the principal surgeon of our county,) to have his opinion, who gave it as his opinion that the bone was implicated, and that an operation would probably be necessary, and thought she had better go to Cincinnati, as the operation would be difficult, attended with dangerous consequences. To this the patient objected, wishing if possible to be treated at home. We watched the progress of the tumor until Jan., 1858, and decided to remove the tumor and save the bone if possible. We now proceeded to remove the tumor, which was as thoroughly done as possible without an incision in the cheek, and then used the cautery very freely; all of which was done without great pain to the patient. In a few weeks subsequent, we were convinced that the malignant character of the disease was not subdued, and that it could only be done by removing that part of the bone implicated. This no one here was willing to undertake. In May, Dr. J. Wise, of Dayton, Ohio, came to our place, with whom I became somewhat acquainted, and learning that he had figured somewhat considerably in surgery, his attention was called to the case, in whose report is the sequel.

This cut represents the tumor and the excised portion of the jaw.

A, is the superior portion of the tumor.

B, The posterior portion of the excised part of the jaw.

C, The base of the jaw.

D, The anterior portion.

This cut represents a vertical longitudinal section.

A, The superior portion of the tumor.

C, The base of the jaw.

D, The anterior portion.

The encroachment of the tumor is shown in the centre of the engraving.

BY DR. J. WISE, DAYTON, OHIO.

About the 20th of May, 1858, while on a medical visit to Hillsboro', Highland county, Ohio, I was informed by Dr. Compton, that a lady of the place by the name of G——, was suffering from the effect of an osseous tumor upon the right side of the inferior maxilla, which he thought would require excision of a part of the jaw. The tumor he believed to be of malignant character, and would soon implicate the largest part of the maxillary bone. The lady was advised by Dr. C. to call upon me which she did, and upon examination I came to the same conclusion with him, that it was a well marked case of osteo-sarcoma of malignant form, and that an operation alone would save the patient. I however, took the dimensions of the tumor, and advised the patient to wait one month, and in the mean time to receive such general treatment as might best prepare the system for an operation, and at that time to operate if the tumor continued to enlarge. Upon our return to Hillsboro' in June, we found the growth of the tumor greater than we expected, it having increased about one-fourth its size in thirty days, and now extended from the right cuspid tooth back about one and a half inches, its width being about two-thirds its length. Being now thoroughly satisfied that an operation was demanded and that delay was dangerous, we proceeded to operate, assisted by Drs. Compton, Homes, and Cresop. Having first satisfied ourselves by auscultation that no lung or cardiac disease existed, Dr. Compton administered chloroform, and we commenced by first making an incision from near the symphysis in a line with the lower edge of the bone back to the angle of the jaw, then dissected up, commencing at the anterior part of the tumor, extending back until the whole was exposed, together with the bone anterior to the tumor, giving room to use the saw. When Dr. Compton excised the bone at the socket of the cuspid tooth (the tooth having been extracted previous to commencing to operate) with a saw, suggested and manufactured by himself, of the mainspring of a watch, which he used in a most admirable manner, severing the bone very quickly without producing the jarring sensation experienced in using the saws generally in use by surgeons; it also cuts very smoothly.

After the bone was severed I continued my dissections after the plan of Dr. Barton of Philadelphia, pulling the bone forward and keeping the facial vein, artery and nerves behind as much as possible, cutting very close to the bone and tumor, until the sound bone was exposed behind the tumor, giving room to use the saw to sever the bone at that point, which was done as expertly as the first by Dr. C. and the operation was soon complete. The loss of blood was small, not exceeding one and a half pounds, only one vessel required a ligature during the whole operation. We closed the wound by the interrupted suture and adhesive plaster, and protected the parts with oiled silk, and kept crushed ice in a bladder constantly applied, and notwithstanding the extreme heat of the season, no pain or inflammation worthy of notice followed, and the wound healed mostly by first intention. The cheek was kept to its place by laying in lint to fill up the part where the tumor was, and in eleven days the patient was allowed to move about the room, and in two weeks to take charge of her household duties. No treatment became necessary except occasional dressing, and the use of light diet. The deformity is very slight, and the patient is entirely well and is able to eat without difficulty.

My opinion is that in all major operations performed in summer, ice should be used; this is the third operation I have performed of removing a part of the maxillary bone, and treated with ice with entire success in each case.

August 6th, 1858.

---

### THE BEST MEANS OF SECURING GOOD TEETH.

BY DR. W. B. INGERSOLL.

(Read at the American Dental Convention.)

This is a question of immense importance, and is upon the lips of the whole community, from childhood to gray hairs.

In discussing questions of this kind we are too apt to take individual cases, and from these establish a precedent, overlooking the primary cause. Too apt to take *that* for absolute which should be considered as secondary. Reasoning from analogy, from comparison, and from what other knowledge we have of the Great Creator. Man was not made an exception in the whole animal creation in this respect that he should be compelled to drop to pieces, inch by inch, casting aside one organ to-day, another to-morrow, alive and yet decaying.

With the hands of death upon us from early youth to hoary age, dragging out a miserable existence in constant pain. Man the only animal, who does or can survive for any considerable length of time after a loss of the teeth.

As well may the King of terrors commence his destructive work upon the limbs or any other part of the body, and let humanity in mass be dismembered, and our whole organic system step out one by one, and fall back to dust again.

If it be true then that originally the dental organs were perfect, is it not equally true, that depraved customs, pernicious habits, and physical transgressions have entailed this lasting evil upon all mankind. Many persons and some scientific men treat this question as though the loss of the teeth was the result of chemical action alone. And, that this chemical action is the fundamental and primary cause.

Many of the experiments which have been made, upon which this hypothesis has been formed were quite unfair.

For instance, to show that the teeth are injured by acids, take a tooth from the jaws, immerse it in strong nitric or hydrochloric acid for one hour, or perhaps for several days; ought such experiments to have a bearing on the question. You may as well bury a man in lake Erie for an hour, then take him out, and because he is a dead man, say—cold water is *deadly* in its effects, and a prudent use of it injurious to health.

In early childhood let us spend less time in *mental* culture, and more in the physical training, develop the muscular system and strengthen the bones. For, it is true with the body as well as the mind,

"Just as the twig is *bent* the tree is inclined."

In youth lay the foundation for *life* and not for *disease*, erect a tabernacle for the mind, which is the man. An immoderate degree of mental cultivation in children is sapping the very vitals of their physical organization.

The mind can not suffer alone, the body must suffer too. The course pursued with children in this country is too much like that which the Irishman said was practised in "swate Ireland." There he says, in building their chimneys they always commence at the top. The question was asked him how they make the first row of bricks stay up. Faith, says Pat, and they put another under it.

If the question be put to the wise and skilful architect, as to the best means of securing a good mechanical structure, would not he say, first of importance is a good quality of materials; wood that is of a healthy growth, firm in its organization, and that, that is of a durable kind, and so with all the materials to be used. And with the teeth, a healthy organization is the first thing to be attained. This of course can not be attained in one year, or in one generation, whether the thing is practicable at all I do not now pretend to answer. It is true that some persons teeth are carried away by external agents, such as acids, and various impurities collected and retained in the mouth. But does that prove conclusively, that these agents are the *absolute* and primary cause of so great a number of persons losing their teeth prematurely. To illustrate more fully, take the hardy oak, let the nut be planted with care, let its germ be nourished in a conservatory, and the rapidity of its growth calls forth the admiration of the amateur and horticulturist, and when its dimensions exceed the capacity of its domicile, take from it its protection and remove entirely the covering, and commit to the tender mercies of a clear blue sky; let the fierce rays of a summer sun come upon it but a few days, and the leaves will wither, its branches droop, and death puts an end to its beauty.

Now what is the cause of this sudden change. Is it not because it has been deprived of those elements which give *consistence* to its wood, *strength* to its fibres, and *vitality* to its organization.

Take another example. It is a very common thing to see members of the same family whose parentage is the same, habits of life and diet the same, each possessed of a strong and vigorous constitution. One will have a *strong*, firm, and durable set of teeth, the other among that class of persons whose teeth are an eye-sore to their parents, a nuisance in society, and a curse to themselves. Again, two persons may start in the world equally the same, so far as human eye can discover both have a good set of teeth, one will take much good care of his teeth, and then perhaps lose them all. The other, almost never any care at all, and keeps them all.

It is a well established fact that in Mechanics, Philosophy, Chemistry, and Dentistry, (all things being equal) like causes produce like effects—(not so altogether in nature.)

Now if any thing is proved by what I have just mentioned, it tends to establish the fact, that with a strong, vigorous, and healthy dental organization, that neither saleratus bread, soda biscuit, acids, alkalies or any other article commonly used as food are sufficient in a direct chemical action to destroy this class of teeth.

Truly without the *aid* of *inspiration*, we can say, " The fathers have eaten sour *grapes*, and the children's teeth are set on edge." A depraved appetite has led us astray, our fashions and diet have furnished death balls instead of bones. *Acids* and *foreign agents prey* upon the imperfectly formed teeth, and have slain their thousands, and weak, frail nature, has lost her ten thousands. It is not necessary that this imperfect organization be external in order to be real, but is frequently occult in its formation, and deceptive in its character.

### CHANCRE ON THE FINGER OF A DENTIST, SUPPOSED TO BE COMMUNICATED FROM THE MOUTH OF A PATIENT.

[From the Boston Medical and Surgical Journal.]

About the 20th of August, 1857, I noticed just above the nail, on the middle finger of the left hand, an oblong, red spot, about the size of a three cent piece. To protect it, I covered it with court plaster. In about a week a vesicle formed, which soon broke, discharged slightly, and was followed by an ulcer about the size of the original red spot. About September 3d, I consulted Dr. A. It was then an indolent ulcer, giving no pain, and he thought it was perhaps derived from a foul tooth in operating. *Treatment*, application of nitrate of silver and compression.

Sept. 8th, ulcer rather worse. There was swelling of a gland in the upper part of the arm, but below the axilla. Consulted Dr. B. The ulcer was then too much irritated to allow him to decide as to its character. *Treatment*, poulticing.

Sept. 12th, another gland just above the elbow became inflamed. The skin over both glands was red and hot, motion of the elbow impeded, ulcer a little extended on one side, and down to the nail. Continued poultices, and take Blancard's pills (iodide of iron) three times a day.

Sept. 26th, Dr. B. being out of town, I consulted Dr. C., who called on Dr. D. to look at it. Dr. D. said it looked like a chancre. Both agreed in a fear that it was malignant. They advised black wash, and doubling the dose of the iodide. Being troubled by this, I consulted Dr. E., who said it was not malignant, but probably like a dissecting-room sore. He advised black wash, and attention to general health.

Sept. 30th, I visited Dr. B. again. He was much dissatisfied with the appearance of the sore, and cauterized it with the acid nitrate of mercury. The application produced extreme pain for about two hours, and at intervals for some hours longer. A dry eschar was produced, from beneath which escaped, on the third or fourth day, a drop of pus. This was the first genuine pus which the sore had produced.

Oct. 14th, eschar separating at the edge. Dr. B. advised a journey and exercise in the open air, as the general health was now much affected.

Oct. 21st, finger somewhat irritable. Eschar loosening. Visited Dr. F., who said that the finger was poisoned, probably by syphilis, as a papular eruption was just appearing. He recommended corrosive sublimate in doses of one-sixteenth of a grain, three times a day, combined with tonics, and good, but plain diet. He sent me to Dr. G., who coincided with him. 23d, Eruption fully developed. *Diagnosis.*—Indurated chancre on the finger, papular eruption, of fully marked syphilitic character. It is useless to pursue the case further, except to say that the progress has fully verified the diagnosis.

My object in giving this account, and in this form, is to call the attention of dentists (and their physicians) to the fact that in the practice of their art they may meet with a similar misfortune, and that its character may not easily be perceived by the most skilful surgeons. In New York, I was assured by Dr. F. (who is a professor, and a very distinguished surgeon), that chancres in the mouth are by no means rare, and perhaps a search would prove them about as plenty in Boston. Should the finger come in contact with such a sore, a hang-nail would give abundant entrance to the poison.

It is almost twenty years since I have practised medicine, and to medical men I do not feel competent to make further comments on the case.

INDIANAPOLIS, AUGUST 1st, 1858.

MESSRS. TAFT & WATT:—At a meeting of the resident dentists of this city, held on the evening of the 18th inst., it was

*Resolved*, That the Dentists of the State generally, be invited to attend a Dental Convention, to be held in this city on the 28th day of December next, for the purpose of forming a State Dental Association.

A circular of invitation will be sent to all practicing Dentists whose names and residences can be ascertained. All other competent practitioners are cordially invited, and earnestly solicited to attend.

J. F. JOHNSTON, Chairman.

G. C. NORTH, Secretary.

## Figures and Tables

**Figure f1:**
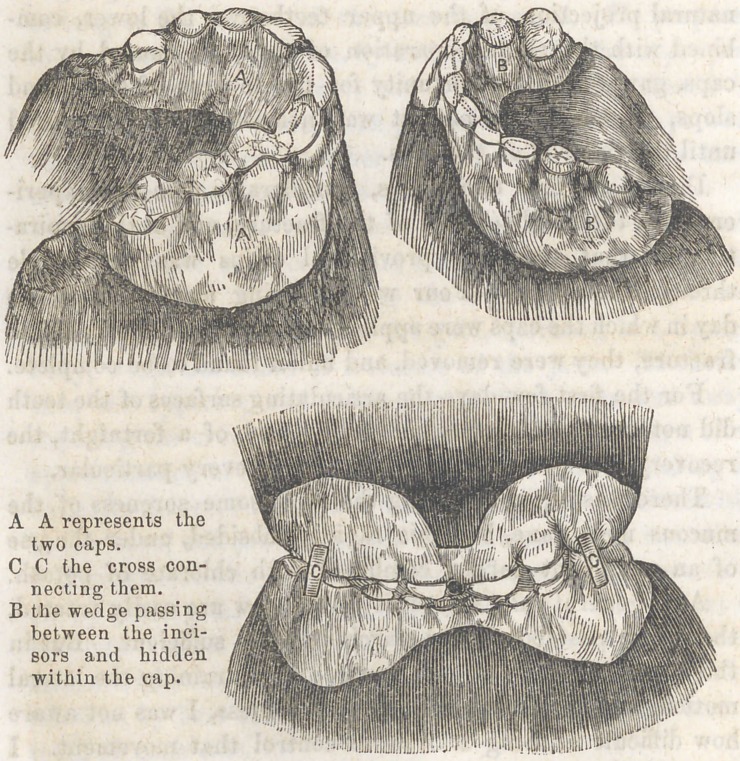


**Figure f2:**
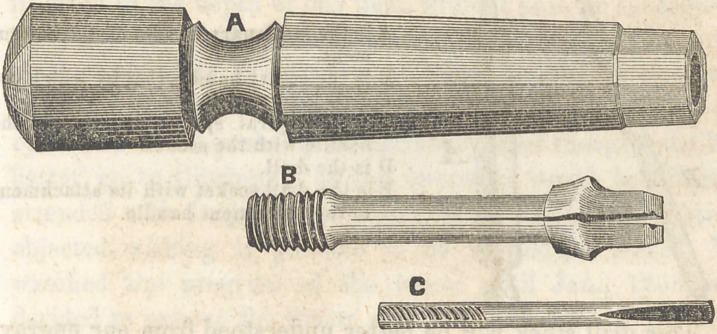


**Figure f3:**
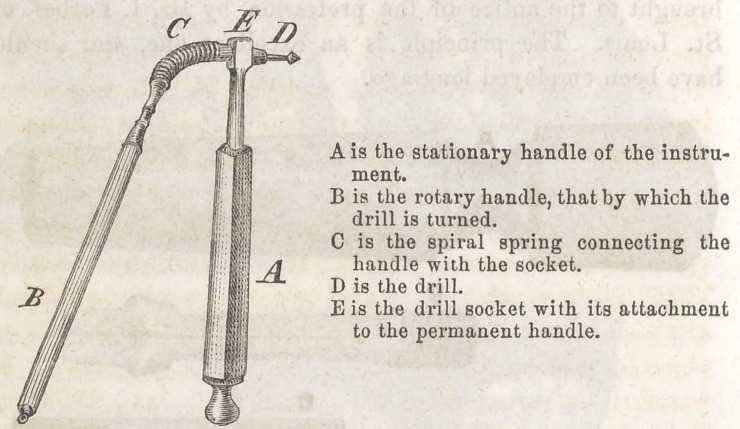


**Figure f4:**
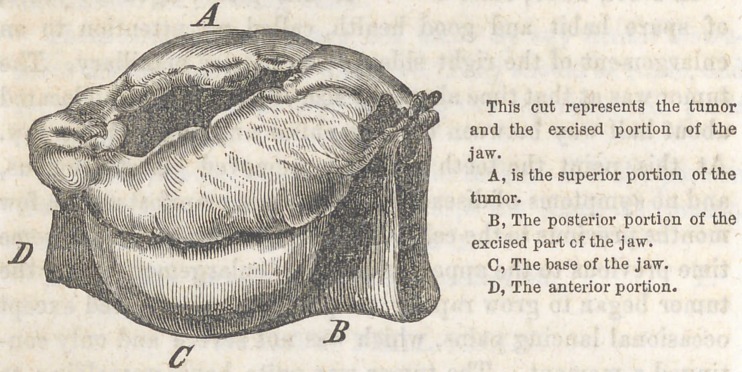


**Figure f5:**